# Decision‐making at the borderline of viability: Who should decide and on what basis?

**DOI:** 10.1111/jpc.13423

**Published:** 2017-02-13

**Authors:** Lynn Gillam, Dominic Wilkinson, Vicki Xafis, David Isaacs

**Affiliations:** ^1^ Children's Bioethics Centre Royal Children's Hospital Melbourne Victoria Australia; ^2^ School of Population and Global Health University of Melbourne Melbourne Victoria Australia; ^3^ Oxford Uehiro Centre for Practical Ethics Oxford United Kingdom; ^4^ John Radcliffe Hospital Oxford United Kingdom; ^5^ Clinical Ethics Sydney Children's Hospital Network Sydney New South Wales Australia; ^6^ Centre for Values Ethics and the Law in Medicine, Sydney Medical School University of Sydney Sydney New South Wales Australia; ^7^ Discipline of Child Health University of Sydney Sydney New South Wales Australia

## Abstract

Parents and medical staff usually agree on the management of preterm labour at borderline viability, when there is a relatively high risk of long‐term neurodevelopmental problems in survivors. If delivery is imminent and parents and staff cannot agree on the best management, however, who should decide what will happen when the baby is delivered? Should the baby be resuscitated? Should intensive care be initiated? Three ethicists, one of whom is also a neonatologist, discuss this complex issue.

## Case History

A 30‐year‐old woman went into threatened labour at 23‐week gestation. She and her husband wanted no active resuscitation and no invasive intervention if the baby was delivered immediately. Labour was successfully halted, and the consultant obstetrician and neonatologist discussed future plans with the parents, who still did not want any invasive ventilation if the mother delivered soon. She went into labour again at 24‐week gestation. After further discussion, the parents agreed to resuscitation and ventilatory support. The baby had a relatively uncomplicated course and survived to discharge, although the long‐term outcome remains uncertain.

On a neonatal ward round, the consultant neonatologist said the prognosis for 24‐week gestation babies had improved to the extent that he was reluctant to allow the parents the option to withhold neonatal intensive care. He stated that if the parents had continued to decline resuscitation he would have acted as the baby's advocate and over‐ruled the parents. The outcome would then depend on the clinical course and on further discussions with the parents. If the baby survived, with or without likely neurodevelopmental problems, and the parents continued to decline to take the baby home, the consultant said he would aim to have the baby adopted.

Neonatal nurses sometimes say that if they were in the same situation as this mother they would remain at home and let the baby die (although that is not necessarily what they would actually do in the circumstances). What should a neonatologist do if both parents decline invasive neonatal intensive care for a baby at the extremes of viability? We asked three experts to comment.

## The Boundaries of the Grey Zone (Dominic Wilkinson, Neonatologist and Ethicist)

While neonatal intensive care is able to save the lives of many infants, some infants still die after a long intensive care stay including multiple painful procedures, while other infants survive long term with substantial morbidity and impairment. For extremely preterm infants, clinicians have often referred to a ‘grey zone’.^1^, ^2^, ^3^, ^4^ Some decisions are black or white: the infant's outlook is either sufficiently poor that resuscitation must not be provided or the outlook is so good that resuscitation is mandatory. Other situations fall somewhere in between and there is acceptance that parents’ wishes are crucial.^5^ In the case example above, clinicians appeared to believe that resuscitation was previously in this grey zone, but a couple of days later had moved into the ‘white zone’, such that non‐resuscitation was no longer appropriate.

### Where are the boundaries of the grey zone?

A large number of publications and guidelines^6^ have provided specific advice on when parental discretion about resuscitation is appropriate. The idea is that an infant's gestational age determines whether or not resuscitation falls within the grey zone. Although there are some differences between these guidelines, there appears to be reasonable international consensus that between 23 weeks and 0 days, and 24 weeks and 6 days, resuscitation may be provided or may be withheld.^6^, ^7^, ^8^ There are no national guidelines in Australia, but previous consensus guidelines developed in NSW/ACT and more recently in Queensland support the appropriateness of providing comfort care (if parents request this after counselling) at 24‐week gestation.^9^, ^10^ That would appear to contradict the approach taken in the case example.

### Has the grey zone changed?

One concern might be that existing guidelines have lagged behind improvements in prognosis. For example, one study from Chicago highlighted that from 1988 to 2008, mortality for infants born prior to 26‐week gestation had fallen from 80 to 28%, however, the boundaries of the grey zone appeared not to have changed.^11^ Over that period, the highest birthweight and gestational age at which resuscitation was not performed (in the absence of congenital abnormalities) had remained static.

Australian and New Zealand Neonatal Network data indicate that, in 1995, 53% of infants born at 24‐week gestation who were admitted to neonatal intensive care units survived to be discharged home.^12^ In the most recent Australian and New Zealand Neonatal Network report, 66% of the same group of infants survived.^13^ These reports do not include neurodevelopmental outcome. Population and centre‐based studies from the USA and UK suggest that approximately 20% of surviving infants born at 24‐week gestation have long‐term severe neurodisability.^14^, ^15^ To put this another way, in the current era in Australia if resuscitation and intensive care is attempted in a tertiary centre, approximately 53% of infants will survive without severe neurodisability.

### Where should the boundaries of the grey zone be?

While gestational‐age‐based guidelines are simple and straightforward for clinicians to apply, there have been a number of critics.^16^, ^17^, ^18^ One concern is that such guidelines can seem worryingly arbitrary. It does not make sense for decision‐making to change at the stroke of midnight, when an infant moves from 23 weeks and 6 days to 24 weeks and 0 days. Moreover, focus on gestational age neglects the significant uncertainty (often ±1 week) that exists about a given infant's gestation.^19^ There are a number of other factors that influence prognosis for an extremely preterm infant including birthweight, sex, the use of antenatal corticosteroids and whether a fetus is a singleton or a multiple.^20^, ^21^ Table 1 illustrates this with two hypothetical extremely preterm infants with identical gestational age. Based on data from a large US cohort study,^20^ the well grown female infant Mary has a chance of surviving without profound impairment that is more than twice that of the growth restricted male infant Mark. It would be bizarre to think that our approach to the ethics of resuscitation/non‐resuscitation should be the same for these two infants with dramatically different chances.

**Table 1:** Two hypothetical 24.3 week infants. Prognosis estimated from the National Institute of Child Health and Human Development online calculator^20^

	Prognostic factors	Estimated chance of either death or profound impairment in ventilated infants (%)
Mary	24.3 weeks, estimated fetal weight 750 g, female singleton, mother given antenatal corticosteroids	33
Mark	24.3 weeks, estimated fetal weight 550 g, male singleton, mother given antenatal corticosteroids	71

Gestational age has been used as a guide to resuscitation decision‐making in preterm infants because it is strongly associated with prognosis.^22^ However, because it is prognosis that is ethically significant, I have argued that we should re‐focus the grey zone specifically on that factor.^22^ Building on existing consensus about the approximate gestational ages where parents should have discretion about resuscitation, and on good quality data about outcome, it is possible to generate a prognosis‐based framework for the grey zone. This framework (Prognosis for Average Gestation Equivalent infant, or PAGE) is illustrated in Table 2. The PAGE framework has been incorporated into recent South Australian guidelines.^23^

**Table 2:** A prognosis‐based framework for decisions around resuscitation and intensive care for extremely premature infants^23^

Estimated chance of poor outcome if intensive treatment is provided (%)†	PAGE	Treatment category	Obstetric management
≤50	≥25 weeks	Usual (life sustaining treatment should usually be provided)	Maternal/fetus focused
50–90	23–24‐week gestation	Optional (life sustaining treatment should be guided by parents’ wishes)	Depends on parents’ wishes
≥90	20–22‐week gestation	Not reasonable (life sustaining treatment should not usually be provided)	Maternal‐focused

† Poor outcome refers to the probability of either death or profound disability (severe, non‐ambulant cerebral palsy or severe cognitive disability).

PAGE, Prognosis for Average Gestation Equivalent infant.

Using the PAGE framework, we can make relevant distinctions between the fictional cases of Mary and Mark. Mary has good prognostic features, meaning that her outlook is more like that of the average 25‐week gestation infant; based on the information available it appears that active resuscitation should occur at birth. Conversely, Mark's prognosis is significantly worse – more like that of an average 23–24‐week infant. Accordingly, treatment appears to lie within the grey zone and non‐resuscitation or resuscitation may be reasonable options.

We do not have enough information about the case given at the start of this article to know whether the decisions made there fit with the framework suggested above. If the infant's prognosis were sufficiently good (such that they had a >50% chance of surviving without profound impairment), the decision to resuscitate against parents’ wishes would have been justified. Conversely, if the infant had other adverse prognostic features (like those applying in Mark's case), the parents should not have been over‐ruled; the mere fact of reaching 24 weeks’ gestation does not mandate a different approach.

One possible concern about the decision is that in another tertiary centre, perhaps even in the same centre if parents had seen a different physician, things would have been different.^24^ There is evidence of dramatic differences in the rates of active resuscitation for extremely premature infants (particularly at 22 and 23‐week gestation) between centres in the USA.^14^ A survey of Australian neonatologists suggested that 94% would be prepared to withhold resuscitation at 24‐week gestation if parents requested this.^25^ However, 6% appeared to indicate that they would go against parental wishes. International studies have suggested even more variability; 40% of US and UK neonatologists were comfortable with not resuscitating a 24‐week infant at parental request^3^, ^26^ while 60% would resuscitate if born in good condition.

The important ethical question raised by this case is one that neonatologists grapple with daily in neonatal units across the country. Improvements in prognosis for extremely premature infants should lead to changes in ethical decision‐making, but it is hard to know where the thresholds for decisions lie. One rational solution would be to re‐focus guidelines for the care of extremely premature infants around *prognosis* rather than just gestational age.^20^ The PAGE framework provides one way of doing this. It is more complicated than standard gestational age‐based guidelines, but has the advantage of being more flexible and transparent than the current approach. It may also help to address troublesome inconsistency between centres and between physicians in their approach to these agonising decisions.

## The Zone of Parental Discretion (Lynn Gillam, Clinical Ethicist)

Would the consultant be ethically justified in intensively treating a 24‐week neonate, against the express wishes of the parents? One way to approach this question is to refer to the Zone of Parental Discretion, an ethical tool intended for use in all sort of situations in which parents and health professionals disagree about the treatment of a child.^27^, ^28^ The fundamental idea of the Zone of Parental Discretion is that parents have a strong ethical (and legal) claim to be the decision‐makers for their child, such that their decisions should not be over‐ridden by others, unless they will cause harm to the child. Taking this approach means thinking first about the parents’ decision, rather than going straight to the question of the clinician's view and what the clinical guidelines say.

What matters most about the parents’ decision is its effect on the child. This is more ethically significant than the parents’ reasons or motivation. Most parents in this sort of situation, instinctively trying to protect their child's life, want everything done. The parents in the above Case History are unusual, which can raise suspicion about their motives, or concern that some past bad experience with disability is overly influencing their decision. Clinicians should of course talk with parents about their wishes for their newborn's medical treatment, to ensure (as much as possible) that they have a good understanding of their baby's medical situation, the decision facing them, and the pathway that lies ahead, whether parents’ first reaction is to treat or not treat. Misunderstandings should be corrected; fears and past experiences should be discussed, to help parents make the most clear‐headed decision they can. However, the decision made by these parents is not inherently suspect. It is worth reflecting that what these parents are saying now is exactly what the neonatologists may be waiting for other parents to ‘come around to’ with other neonates.

Let us assume that after discussion along the lines of the above, the parents still do not want resuscitation and intensive treatment for their newborn. Now the key ethical question is whether their decision would constitute ‘causing harm’ to their child. ‘Causing harm’ is more than just failing to maximise the child's well‐being. It involves a significant set‐back to the child's interests, which can be predicted with a reasonable degree of probability. Would this baby's interests be significantly set back by taking the parents’ preferred palliative approach? Trying to answer this means comparing the two possible pathways ahead for this baby. The palliative pathway is predictably very short, but with a low level of suffering. Without active intensive care, the baby will almost certainly die within a short period of time. With good palliative care, he will die in his parents’ arms, without experiencing any suffering. The active treatment pathway is much less predictable, especially in the long term. It will start with many invasive procedures in a neonatal intensive care unit – discomfort, pain, perhaps on many occasions, lots of people coming and going, continuous noise and lights, limited physical contact with mother and father: all unavoidable side effects of medical treatment aimed at sustaining life and maximising the chances of a good long‐term outcome. This pathway may branch off in many different directions, at different stages. The baby may be in neonatal intensive care unit (NICU) for some weeks, but deteriorate and die, despite all efforts to keep him alive. Alternatively, after some months in NICU, he may be well enough to go to the ward, and later home, with some on‐going problems and deficits, but with no significant physical or cognitive disabilities. In addition, there are many other possible outcomes somewhere in between.

Is the palliative pathway ***so much worse*** for the baby than the intensive care pathway, that it would constitute ***causing harm*** to the baby? This is not an easy question to answer. Firstly, it involves trying to weigh up and balance the different and competing interests of the baby. Feeling comfortable, safe and pain‐free in short term are all very important interests, but compete with the interest in having a longer life, perhaps in adulthood, with all the life experiences that may bring. The weighing up is infinitely complicated by uncertainty, and the available evidence does not provide many help in prognosticating for individuals, especially over such a long time‐span. In addition, this is not just a matter of making calculations about facts and probabilities; it is also about values. The underlying questions are these: what sort of life is a good life, and how much burden and risk is it reasonable to impose on a baby in the hope of achieving this? In this situation, parents and the neonatologist have different answers to these questions, based on different values. Both answers may be reasonable, and neither one incorrect.

An important further consideration to take into account is that providing full active treatment in this situation involves doing what the parents do not want, and most likely believe is not best for their baby. They will have to try to meet his needs and provide good experiences for him in NICU and for as long as he survives, while in a negative state of mind. If he survives long term, but with significant disabilities, he will be very dependent on his parents not just for his physical care, but also for the emotional and social environment on which his chances of a happy, meaningful life will depend. In short, it is important to be precise about the pathways we are comparing: palliative care with parents ‘on‐board’, and intensive care with parents not on‐board.

It is easy to say that if his parents do not want him, he can just be adopted. The emotional and practical reality will be much more complex. His parents will not necessarily be willing to have him adopted. It cannot be assumed that they are more worried about themselves than about him. Perhaps they might agree to fostering if he survives to discharge, but that will mean a search for foster parents willing and able to look after this child. A number of short‐term placements are quite foreseeable. The child may end up in the care of a series of strangers, lose contact with siblings and extended family, all while coping with disability and on‐going medical treatment. The possibility of doing unintended damage by going against the parents’ decision must be borne in mind.

What about a trial of treatment for a specified time, to see how he goes? Since withdrawal of intensive care treatment is ethically equivalent to withholding it, there is no ethical obligation to continue, just because it has been started. If ultimately it is decided that the parents’ wishes not to pursue life‐prolonging treatment should be accepted, then the intensive treatment can simply be stopped. However, it is important to be aware that there are costs to buying time in this way. The baby is having all the negative experiences that being treated in NICU entails, with angry or distressed parents by the bedside, or indeed hardly by the bedside at all. In addition, parents and staff may find withdrawal of ventilation more emotionally fraught than withholding it, even though they are in logical ethical terms equivalent.

In essence, the bottom‐line question is this: ‘Are we ***sure enough*** that the parents’ decision is ***bad enough*** for the child that over‐riding it is warranted?’ My own answer (acknowledging the lack of detailed information about the case) is ‘No’.

## Seeking Clarity Where No Clarity Exists: Should Clinicians Over‐ride Parental Requests Not to Provide Life Sustaining Treatment for Babies at the Borderline of Viability? (Vicki Xafis, Clinical Ethicist)

In cases where genuine medical uncertainty exists as to a premature baby's chances of survival or severe neurodevelopmental impairment, parents’ requests to forego treatment for such premature infants should not be over‐ridden by clinicians who believe they have a duty to advocate for the baby.

### Who should decide?

Numerous models of shared decision‐making exist.^29^, ^30^, ^31^ In the paediatric context, where the child is unable to participate in the process, there are legitimate concerns about the ability to focus exclusively on the child's interests given the existence of clinician and parental interests,^32^, ^33^ which may at times conflict with the child's interests. Nevertheless, there is recognised value in clinicians and parents engaging in shared decision‐making.^30^, ^34^ Ultimately, however, parents are acknowledged, ethically and legally, as having parental authority to make medical decisions for their children even if they are not *optimal* and even if clinicians *are not always in full agreement*.^28^ In practice, parents are not always included in decisions about their child,^35^, ^36^, ^37^, ^38^, ^39^ perhaps due to time constraints at the time decisions are made^40^ as well as differences in practice across different centres.^41^

### When are differing views of concern?

When clinician and parental views diverge significantly, the benefits and harms of the preferred treatment decisions are assessed via consideration of the Best Interests Principle or the Harm Principle, which enable us to determine and weigh up the benefits and harms of the proposed courses of action for the child. Some ethicists favour the Harm Principle, according to which parental authority should only ever be curtailed when parental decisions will cause significant harm to a child.^28^, ^42^ In cases where parental preferences for treatment raise serious concerns, it is the clinician's duty to advocate for and protect the child by over‐riding parental decisions or seeking the court's assistance in determining who should make the decision.^43^

I argue that over‐riding parental decisions to forego treatment for babies at the borderline of viability where there is doubt about the prognosis in an effort to advocate for the baby cannot reasonably be justified and that such efforts could have disastrous effects.

### Can the normative principles of best interests or harm be applied in cases of great medical uncertainty?

While principles such as those previously mentioned may be appropriate in the consideration of most paediatric decisions where, guided by clinical data, it is possible to ascertain with some clarity the potential benefits and/or harms of treatment decisions, they cannot apply in cases where there is no definitive evidence of whether a baby will/will not survive or whether s/he will/will not suffer devastating neurodevelopmental outcomes. Over‐riding parental requests to forego treatment in these circumstances cannot be justified by outcome data given their variability^44^, ^45^, ^46^, ^47^ and their inapplicability to specific babies,^44^, ^47^ a fact that some parents also recognise.^48^

When discussing infants at the borderline of viability, Brunkhorst and colleagues suggest that ‘Dying is usually not in an infant’s best interest’ and that ‘It is hard to argue that death without a proper chance at life is ever in the best interest of an infant.’^48^ On an emotional level, these statements are welcome. In reality, however, for some babies who may survive against all odds, their short or longer lives consist in innumerable hospital admissions, painful treatments, great discomfort, perhaps numerous perilous moments that appear to approach death, and even death itself. Therefore, in such cases, we cannot claim that the mere fact of being alive is a benefit and in the interests of the child. Similarly, it is difficult in cases where there is genuine medical uncertainty to argue that parents’ decisions to withhold treatments are harmful to the child given that the baby's future is filled with unknowns. Decisions to provide treatments may, in fact, prove to be harmful for all involved; this, once again, is unknown and unknowable in advance.

### Differences in clinician and parental decision‐making

Decisions for babies at the borderline of viability are guided by slightly different resources, tools, reserves and outlooks depending on who is making the decision. This fact is rarely taken into account but is relevant to the appropriateness of decisions made.

For clinicians, prominently assisting with decision‐making are:

- **Technical components**, such as outcome data,^20^, ^49^ evidence‐based prognostic tools, such as the NICHD outcomes calculator, which provides a range of potential outcomes rather than individual outcomes^50^ and clinical guidelines^6^, ^23^
- **Clinical components**, such as the actual presentation of the baby at birth which impacts on decisions regarding resuscitation^3^ as well as results from tests administered to the baby
- **Theoretical components**, such as local or international guidelines which take into account ethically and legally relevant facts critical to such decisions^40^, ^51^

Personal beliefs and views no doubt also influence clinicians’ interpretations of clinical facts and their decisions as do adopted practices in various centres. Furthermore, while parental wishes and values are considered by clinicians, their focus is primarily on doing what is best for the baby. Despite the resources and the clinical expertise and experience, clinicians can sometimes struggle to determine what the best and most ethically appropriate course of action is for these babies.

For parents, the landscape is markedly different. Outcome and prognostic data are important to most parents even though some parents assert that such data played a secondary role in the decisions they made for their child.^44^, ^47^ Furthermore, the role such data play in parental decision‐making is affected by the manner with which they are presented^52^ as well as broader clinician/parental communicative interactions.^53^

Some common defining features of parental decision‐making include the fact that:

- Parents are emotionally and psychologically invested in any decision made
- Parents frequently lack the technical expertise to assess complex clinical information
- Parental decisions must be weighed up against other important values defining their lives
- Any decision will comprise an important aspect of their individual and family make‐up for the rest of their lives

Powerful parental instincts usually prompt parents to advocate for treatments no matter how slim the chances may appear, sometimes even against clinicians’ advice.^50^, ^54^ More rarely, after consideration of the clinical facts and other important values and beliefs, parents may choose to forego treatments for their child in the face of such great uncertainty. Influencing such decisions may also be parents’ psychological and emotional inability to withdraw treatments at a later stage^29^ following a recommendation from clinicians to commence a treatment trial. Furthermore, weighing into parental decisions, and possibly sometimes overlooked, is that parents are tasked to make decisions about their baby within an intricate network of relational moral obligations.^55^

In cases of borderline viability, clinicians may sometimes feel compelled to advocate for the neonate and provide treatment against parental wishes to forego treatments. Such decisions are not ethically justifiable if it is impossible to weigh up the potential harms to the child or to consider what is in the best interests of the child due to a lack of prognostic certainty. What *is* certain is that no parental decision to forego treatment and forego the dreams of including their baby in their lives comes easily.^27^, ^56^, ^57^ Clinicians will perhaps always remember the individual ‘case’ where they attempted to advocate for the child but the impact of their specific decision for a specific child will fade as they go on to make decisions about hundreds of other children throughout their medical careers. However, the child and the parents of the child for whom clinicians advocated against parental wishes will always bear the potential burdens of their intervention for the rest of their lives.

## Conclusion

The aim of this article was to provide a brief ethical analysis of a clinically and ethically complex issue. Ideally, decisions about whether or not to resuscitate a baby at the extreme of viability should be shared decisions, made by both parents with unbiased information from expert clinicians.

The three ethicists have taken different approaches but there are common threads. We need to ask the right questions to ascertain whether parental decisions may cause harm; adoption of the right clinical framework will assist in determining the ethical acceptability of such parental decisions; but where there is genuine uncertainty and the outcome could be either of the two extremes, the harm and best interests principles cannot guide such decisions, so parental decisions should not be over‐ruled.

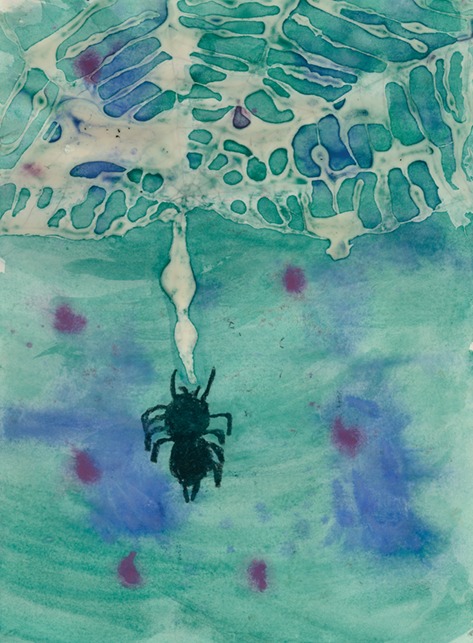

Baby spider hanging by a thread by Skyla Kersting (age 8) from Operation Art 2016
